# Differential impact of SARS-CoV-2 infection during different outbreak periods on incident diabetes in Japan: a matched cohort study utilizing health insurance claims

**DOI:** 10.1265/ehpm.24-00191

**Published:** 2024-10-03

**Authors:** Akiko Matsumoto, Sachiko Kodera, Tatsuya Matsuura, Yoko Takayama, Yuya Yamada, Akimasa Hirata

**Affiliations:** 1 Department of Social and Environmental Medicine, Saga University, Saga 849-8501, Japan; 2 Center of Biomedical Physics and Information Technology, Nagoya Institute of Technology, Nagoya 466-8555, Japan; 3 Japan System Techniques Co., Ltd, 2-16-2 Konan, Minato-ku, Tokyo 108-8288, Japan

**Keywords:** COVID-19, Diabetes, Health insurance system, Hazard ratio

## Abstract

**Background:**

An increased risk of diabetes after COVID-19 exposure has been reported in Caucasians during the early phase of the pandemic, but the effects across viral variants and in non-Caucasians have not been evaluated.

**Methods:**

To address this gap, survival analyses were performed for five outbreak periods. From an anonymized health insurance database REZULT for the employees and their dependents of large companies or government agencies in Japan, 5 matched cohorts were generated based on age, sex, area of residence (47 prefectures), and 7 ranges of medical bills (COVID-19 exposed:unexposed = 1:4). Observation of each matching group began on the same day. Incident diabetes type 1 (T1D) and type 2 (T2D) were defined as the first claim during the target period, including at least 1 year before the start of observation.

**Results:**

T1D accounted for 0.8% of incident diabetes after the first COVID-19 exposure, similar to the non-exposed cohort. Most T2D in the COVID-19 cohort was observed within a few weeks. After further adjustment for the number of days from the start of observation to hospitalization (a time-dependent variable), the hazard ratio for incident T2D ranged from 14.1 to 20.0, with 95% confidence intervals (95%CI) of 8.7 to 32.0, during the 2-month follow-ups from the original strain outbreak to the Delta variant outbreak (by September 2021), and decreased to 2.0, with a 95%CI of 1.6 to 2.5, during the Omicron outbreak (by March 2022). No association was found during the BA.4/5 outbreak (until September 2022). Males had a higher risk, and the trend toward higher risk in older age groups was inconsistent across the periods.

**Conclusions:**

Our large dataset, covering 2019–2023, reports for the first time the impact of COVID-19 on incident diabetes in non-Caucasians. The risk intensity and attributes of post-COVID-19 T2D were inconsistent across outbreak periods, suggesting diverse biological effects of different SARS-CoV-2 variants.

**Supplementary information:**

The online version contains supplementary material available at https://doi.org/10.1265/ehpm.24-00191.

## Background

The after-effects of coronavirus disease 2019 (COVID-19) are one of the major concerns [1]. Among others, an increased risk of diabetes after COVID-19 exposure has been reported for multiple countries, however, most subjects in large cohorts are Caucasian [2–8] with inconsistent focus, i.e., type 1 diabetes (T1D) [4, 5], young age diabetes [2], diabetes of any type [6–8], type 2 diabetes (T2D) (comparison with influenza control) [3]. Considering the possibility of race-specific effects, as reported for COVID-19 incidence for Japanese [9, 10], such studies are needed for non-Caucasians. Moreover, most observation began in 2020 to early 2021. Large-scale cohort studies of various viral variants are currently lacking. To evaluate the incidence of COVID-19-induced diabetes throughout the COVID-19 pandemic, we evaluated five matched cohorts from waves 1 to 7 using a health insurance claims database representing approximately 2.4% of the Japanese population.

## Methods

The anonymized database REZULT (https://www.jastlab.jast.jp/rezult_data/) was provided by Japan System Techniques, Inc., on behalf of the Japanese government. This database contains information on the health insurance claims of employees of large companies or government agencies and their dependents, including T1D and T2D according to the International Classification of Diseases, Tenth Revision, Clinical Modification, codes of E10 and E11, respectively. According to the predominant SARS-CoV-2 strain during outbreaks (Fig. S1a), the seven waves were divided into five periods: (I) Waves 1–3 with Wuhan strain (April 2020 to March 2021), (II) Wave 4 with Alpha variant (April to May 2021), (III) Wave 5 with Delta variant (July to September 2021), (IV) Wave 6 with Omicron variant (January to March 2022), and (V) Wave 7 with BA.4/5 variant (July to September 2022).

**Fig. 1:**
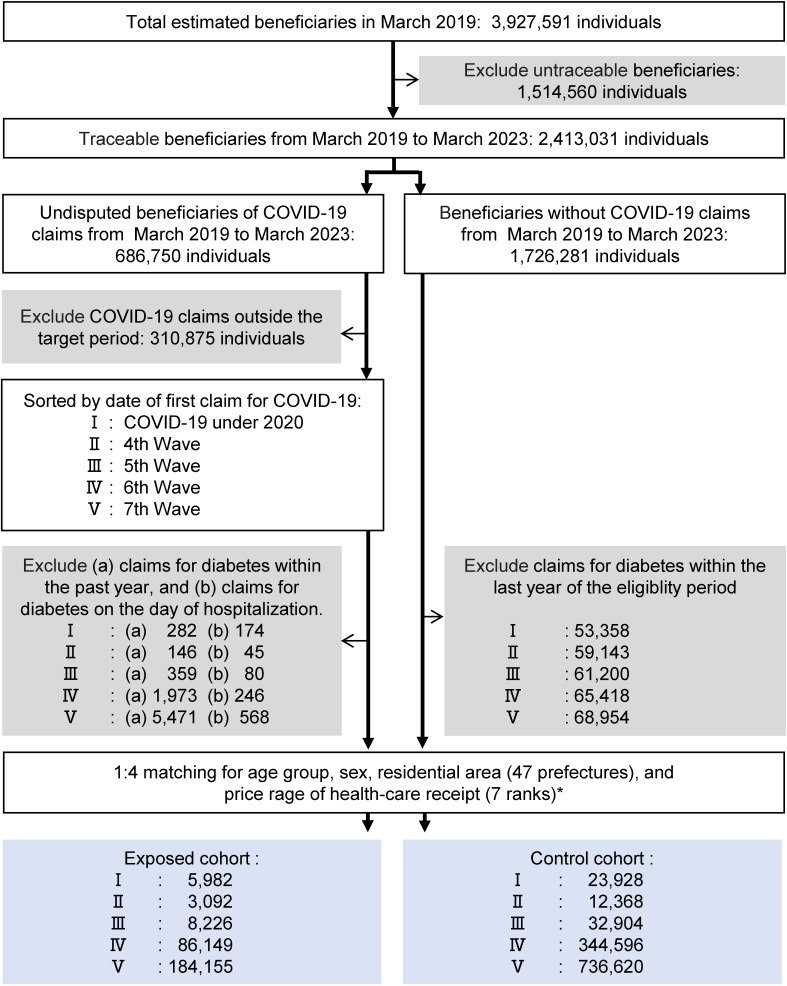
Flowchart extracting the 1:4 matched cohort. The matched cohorts were extracted from a health insurance claims database of employees of large companies or government agencies and their dependents in Japan. *The price range of healthcare receipt was divided into 7 ranges: annual amount <50,001 yen, 50,001–100,000 yen, 100,001–150,000 yen, 150,001–200,000 yen, 200,001–500,000 yen, 500,001–1,000,000 yen, and >1,000,000 yen.

As shown in Fig. 1, of the 4 million beneficiaries in March 2019, 2,413,031 were identified as traceable between March 2019 and March 2023, based on claims in both 2019–2020 and 2023. The first claims for COVID-19 diagnosed by a positive nucleic acid amplification test or antigen test during the target periods were extracted (N = 375,875) from the entire 34-month period (N = 686,750). After excluding diabetes claims within one year before observation, and diabetes claims on the day of hospitalization to account for the spurious association due to the increased likelihood of diagnosis by hospitalization, 287,604 COVID-19 claims were successfully matched to 1,150,416 non-exposed beneficiaries by age, sex, area of residence (47 prefectures), and the 7 ranges of medical bills incurred in the preceding year. For the cohorts IV and V, the follow-up period was truncated to March 2023.

The Cox proportional hazard ratio (HR) for incident diabetes by first exposure to COVID-19 was calculated using IBM SPSS Statistics version 29 (IBM Corp., Armonk, NY, USA), with a primary observation period of 2 months to maintain proportional hazards (Figs. S2 and S3). Observation of each 1:4 matching group began on the same day. Cohort-matching covariates and a time-dependent covariate, the number of days from the start of the observation to hospital admission, were modeled.

## Results

**Fig. 2:**
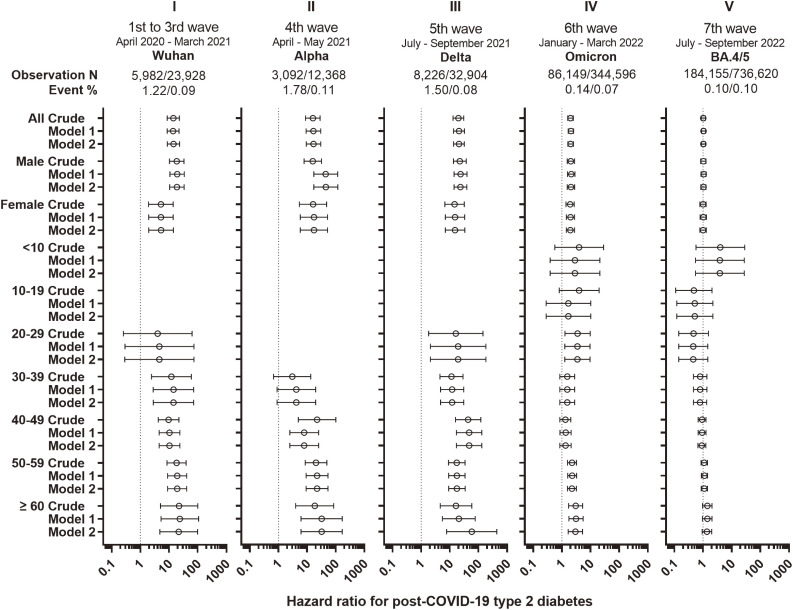
Hazard ratio for incident type 2 diabetes after the first COVID-19. The incidence of diabetes after COVID-19 was subjected to survival analyses for 5 outbreak periods utilizing the Japanese health insurance claims database. Matched cohorts were generated based on age group, sex, area of residence (47 prefectures), and the 7 ranges of medical bill amounts (COVID-19 exposed:unexposed = 1:4) for the following 5 observation periods. To maintain proportional hazards, estimates were made for 2-month periods. Model 1 includes age group, sex, area of residence, and price range of health care use as covariates. Model 2 includes an additional time-dependent covariate, days to hospitalization.

T2D accounted for 99.2% of diabetes (554/558) (Tables S1 and 1), with two cases having a history of both T1D and T2D. Most of the excess incidence of T2D in the exposed cohorts compared with that in the non-exposed cohort occurred within a few weeks (Fig. S3). T1D was similarly observed in the exposed and non-exposed cohorts (p for Fisher’s exact test >0.15 and >0.49 for the 2-month and 1-year follow-up, respectively), whereas T2D was highly prevalent in the exposed cohorts (Tables 1 and S2 and Fig. 2). As shown in Fig. 2 and Table S3, the HRs (95% confidence intervals, 95%CI) in fully adjusted models for T2D were 14.2 (8.7–23.1), 16.7 (9.3–30.1), and 20.8 (13.5–32.0) in periods I, II, and III, respectively (2-month observation, model 2), whereas the value decreased to 2.0 (1.6–2.5) in the Omicron period (IV) and to 1.0 (0.9–1.2) in the final period (V). Males showed higher HRs than females at different intensities, and the age effect was inconsistent, showing a trend from younger to older in the early phase and no apparent trend in III and later. The trend was the same at the 1-year follow-up with lower HRs (3.5–4.7 in I–III, 1.3 in IV, and 1.1 in V) (Table S4).

**Table 1:** Incident type 2 diabetes in COVID-19 exposed and non-exposed cohorts during 2-month follow-up.

	**I**		**II**		**III**		**IV**		**V**	

	**2020 (April 2020–March 2021)**		**4th (April–May 2021)**		**5th (July–September 2021)**		**6th (January–March 2022)**		**7th (July–September 2022)**	
	**Exposed**	**Non-exposed**	**Exposed**	**Non-exposed**	**Exposed**	**Non-exposed**	**Exposed**	**Non-exposed**	**Exposed**	**Non-exposed**

	**Cases/observations (%)**		**Cases/observations (%)**		**Cases/observations (%)**		**Cases/observations (%)**		**Cases/observations (%)**	
All	73/5,982 (1.22)	21/23,928 (0.09)	55/3,092 (1.78)	14/12,368 (0.11)	123/8,226 (1.50)	25/32,904 (0.08)	118/86,149 (0.14)	240/344,596 (0.07)	187/184,155 (0.10)	733/736,620 (0.10)
<10 y.o.	0/845 (0.00)	0/3,380 (0.00)	0/340 (0.00)	0/1,360 (0.00)	0/944 (0.00)	0/3,776 (0.00)	1/12,194 (0.01)	2/48,776 (0.00)	2/12,475 (0.02)	1/49,900 (0.00)
10–19	0/645 (0.00)	0/2,580 (0.00)	1/374 (0.27)	0/1,496 (0.00)	0/1,418 (0.00)	0/5,672 (0.00)	3/17,312 (0.02)	3/69,248 (0.00)	2/33,029 (0.01)	16/132,116 (0.01)
20–29	1/857 (0.12)	1/3,428 (0.03)	1/433 (0.23)	0/1,732 (0.00)	4/1,299 (0.31)	1/5,196 (0.02)	7/9,199 (0.08)	8/36,796 (0.02)	3/20,250 (0.01)	26/81,000 (0.03)
30–39	6/1,097 (0.55)	2/4,388 (0.05)	3/556 (0.54)	4/2,224 (0.18)	17/1,523 (1.12)	6/6,092 (0.10)	15/17,975 (0.08)	39/71,900 (0.05)	16/32,571 (0.05)	81/130,284 (0.06)
40–49	19/1,055 (1.80)	8/4,220 (0.19)	11/538 (2.04)	2/2,152 (0.09)	43/1,550 (2.77)	4/6,200 (0.06)	25/16,741 (0.15)	79/66,964 (0.12)	48/42,023 (0.11)	216/168,092 (0.13)
50–59	36/1,143 (3.15)	8/4,572 (0.17)	30/637 (4.71)	6/2,548 (0.24)	47/1,224 (3.84)	11/4,896 (0.22)	46/9,713 (0.47)	81/38,852 (0.21)	80/33,541 (0.24)	291/134,164 (0.22)
≥60	11/340 (3.24)	2/1,360 (0.15)	9/214 (4.21)	2/856 (0.23)	12/268 (4.48)	3/1,072 (0.28)	21/3,015 (0.70)	28/12,060 (0.23)	36/10,266 (0.35)	102/41,064 (0.25)

Males	64/3,202 (2.00)	14/12,808 (0.11)	39/1,597 (2.44)	10/6,388 (0.16)	94/4,355 (2.16)	17/17,420 (0.10)	67/40,355 (0.17)	130/161,420 (0.08)	114/85,355 (0.13)	443/341,420 (0.13)
<10 y.o.	0/464 (0.00)	0/1,856 (0.00)	0/183 (0.00)	0/732 (0.00)	0/545 (0.00)	0/2,180 (0.00)	0/6,123 (0.00)	0/24,492 (0.00)	2/6,139 (0.03)	0/24,556 (0.00)
10–19	0/345 (0.00)	0/1,380 (0.00)	1/213 (0.47)	0/852 (0.00)	0/763 (0.00)	0/3,052 (0.00)	1/9,542 (0.01)	3/38,168 (0.01)	1/15,912 (0.01)	5/63,648 (0.01)
20–29	0/410 (0.00)	0/1,640 (0.00)	0/200 (0.00)	0/800 (0.00)	3/653 (0.46)	1/2,612 (0.04)	2/4,263 (0.05)	6/17,052 (0.04)	2/9,043 (0.02)	19/36,172 (0.05)
30–39	6/550 (1.09)	1/2,200 (0.05)	1/256 (0.39)	1/1,024 (0.10)	12/752 (1.60)	3/3,008 (0.10)	7/7,437 (0.09)	17/29,748 (0.06)	4/13,895 (0.03)	43/55,580 (0.08)
40–49	15/551 (2.72)	6/2,204 (0.27)	7/267 (2.62)	2/1,068 (0.19)	38/792 (4.80)	3/3,168 (0.09)	14/6,762 (0.21)	41/27,048 (0.15)	30/18,507 (0.16)	124/74,028 (0.17)
50–59	33/661 (4.99)	7/2,644 (0.26)	25/351 (7.12)	5/1,404 (0.36)	31/677 (4.58)	8/2,708 (0.30)	27/4,579 (0.59)	43/18,316 (0.23)	50/16,288 (0.31)	189/65,152 (0.29)
≥60	10/221 (4.52)	0/884 (0.00)	5/127 (3.94)	2/508 (0.39)	10/173 (5.78)	2/692 (0.29)	16/1,649 (0.97)	20/6,596 (0.30)	25/5,571 (0.45)	63/22,284 (0.28)

Females	9/2,780 (0.32)	7/11,120 (0.06)	16/1,495 (1.07)	4/5,980 (0.07)	29/3,871 (0.75)	8/15,484 (0.05)	51/45,794 (0.11)	110/183,176 (0.06)	73/98,800 (0.07)	290/395,200 (0.07)
<10 y.o.	0/381 (0.00)	0/1,524 (0.00)	0/157 (0.00)	0/628 (0.00)	0/399 (0.00)	0/1,596 (0.00)	1/6,071 (0.02)	2/24,284 (0.01)	0/6,336 (0.00)	1/25,344 (0.00)
10–19	0/300 (0.00)	0/1,200 (0.00)	0/161 (0.00)	0/644 (0.00)	0/655 (0.00)	0/2,620 (0.00)	2/7,770 (0.03)	0/31,080 (0.00)	1/17,117 (0.01)	11/68,468 (0.02)
20–29	1/447 (0.22)	1/1,788 (0.06)	1/233 (0.43)	0/932 (0.00)	1/646 (0.15)	0/2,584 (0.00)	5/4,936 (0.10)	2/19,744 (0.01)	1/11,207 (0.01)	7/44,828 (0.02)
30–39	0/547 (0.00)	1/2,188 (0.05)	2/300 (0.67)	3/1,200 (0.25)	5/771 (0.65)	3/3,084 (0.10)	8/10,538 (0.08)	22/42,152 (0.05)	12/18,676 (0.06)	38/74,704 (0.05)
40–49	4/504 (0.79)	2/2,016 (0.10)	4/271 (1.48)	0/1,084 (0.00)	5/758 (0.66)	1/3,032 (0.03)	11/9,979 (0.11)	38/39,916 (0.10)	18/23,516 (0.08)	92/94,064 (0.10)
50–59	3/482 (0.62)	1/1,928 (0.05)	5/286 (1.75)	1/1,144 (0.09)	16/547 (2.93)	3/2,188 (0.14)	19/5,134 (0.37)	38/20,536 (0.19)	30/17,253 (0.17)	102/69,012 (0.15)
≥60	1/119 (0.84)	2/476 (0.42)	4/87 (4.60)	0/348 (0.00)	2/95 (2.11)	1/380 (0.26)	5/1,366 (0.37)	8/5,464 (0.15)	11/4,695 (0.23)	39/18,780 (0.21)

## Discussion

The current study is the first to report on diabetes after COVID-19, with a large dataset covering 2019–2023 for non-Caucasians. The effects of COVID-19 on incident T1D were not detected, whereas increased HRs for T2D were evident. HRs during the 2-month observation period were high in the early phase ranging from 14.2 to 20.8, and decreased dramatically in the omicron to 2.0 and BA.4/5 to 1.0.

Previous studies of diabetes of any type in UK residents enrolled in 2020 [6] or US veterans enrolled until March [7] or September 2021 [8] are comparable to our cohorts I–III. Our study yielded higher estimates, suggesting a race-specific susceptibility as demonstrated for incident COVID-19 [9, 10]. For example, the UK study reported a rate ratio of 1.4 at 140 days mean follow-up for Caucasians [6], and US veteran studies reported an odds ratio of 2.56 for males at 120 days [7] and HR of 1.46 during 1 year follow-up [8]. These studies report higher susceptibility in non-Caucasians, e.g., an odds ratio of 1.6 in black males vs. non-black males [7].

Although ethnic- and variant-specific biological mechanisms are not well-understood, several explanations for COVID-19-induced diabetes have been proposed, such as impaired insulin secretion and glucose disposal [11]. For example, the autopsy of COVID-19 cases showed that SARS-CoV-2 caused the transdifferentiation of pancreatic beta-cells into alpha and acinar cells, resulting in reduced insulin secretion [12]. Transient hyperglycemia due to inflammation has also been suggested [11]. In our study, transient hyperglycemia induced by a stress response to inflammation may have been counted as diabetes. A major limitation of this study is the uncertainty of whether “diabetes” persists and continues to affect quality of life. However, when comparing influenza and COVID-19 infections of similar severity, the incidence of diabetes was reported to be approximately 1.5 times higher in COVID-19 cases [3], suggesting a unique effect of the SARS-CoV-2 virus.

The effect of COVID-19 beyond Omicron (IV and later) is a new discovery. In these periods, the number of fully vaccinated people increased, and the number of COVID-19 cases exploded because the Japanese government did not declare a state of emergency. The severity of the disease has decreased, which is thought to be the main reason for the decrease in HRs to 2.0 and less. However, the possibility of overestimation should be considered as there may have been an increase in cases where individuals tested antigen and did not seek medical attention, especially for mild cases. This may have resulted in the COVID-19 exposure group having a relatively higher severity than in reality.

Although this is the first large non-Caucasian cohort, a limitation of this study is insufficient sample size to observe the impact on T1D due to its low prevalence in Japan. The lack of linkage of healthcare claims to vaccination status is another limitation, as cohorts exposed to COVID-19 are expected to be less vaccinated than unexposed cohorts [13]. Given the vaccination status in Japan (Fig. S1b), the risk may have been overestimated in phases III and IV. In addition, although we attempted to control unmeasured confounders using a matching strategy that included 47 prefectures and 7 price bands of healthcare expenditures, the HRs might be overestimated by residual confounders such as obesity. The limitation in generalizability should also be addressed, as the database does not include insurance associations for employees of small enterprises or sole proprietors. Finally, the possibility that undiagnosed diabetes was the reason for the COVID-19 event should be considered. This reversal of causation, however, is unlikely to explain the majority of T2D cases after COVID-19, because annual diabetes screening is mandatory for Japanese after the age of 40, and the screening rate is approximately 80% in the corresponding population [14].

## Conclusions

From an anonymized health insurance database representing 2.4% of the Japanese population, 5 finely matched cohorts were examined for the effect of first exposure to COVID-19 on incident diabetes during the COVID-19 outbreaks from 2019 to 2023. The effect of COVID-19 on incident T1D was not well assessed due to insufficient sample size, whereas T2D was observed to increase rapidly within a few weeks. The hazard ratio was high in the early phase, from 2019 to 2021, and decreased dramatically after the Omicron outbreak. Comprehensive multi-level analyses of different variants in different regions are still needed, along with elucidation of biological mechanisms.

## Abbreviations

COVID-19: coronavirus disease 2019
T1D: type 1 diabetes
T2D: type 2 diabetes
HR: hazard ratio

## Supplementary information

**Additional file 1:** **Figure S1.** Summary of publicly available statistics in Japan on (a) sequences by viral variant of SARS-CoV-2 and (b) rate of COVID-19 vaccination. **Figure S2.** Plots of log (−log (day of diabetes-free survival)) versus log (diabetes-free survival ratio). **Figure S3.** Type 2 diabetes-free survival curves by COVID-19 exposure stratified by (a) sex and (b) age. **Table S1.** Incident type 1 diabetes in COVID-19 exposed and non-exposed cohorts during 2-month follow-up. **Table S2.** Incident type 2 diabetes in COVID-19 exposed and non-exposed cohorts during 1-year follow-up. **Table S3.** Hazard ratio estimation for type 2 diabetes by exposure to the first COVID-19 with 2-month follow-up. **Table S4.** Hazard ratio estimation for type 2 diabetes by exposure to the first COVID-19 with 1-year follow-up.

## Declarations

### Availability of data and material

We are not authorized to publish the database due to data security and privacy reasons.

## References

[1] Mahase E. Covid-19: What do we know about “long covid”? BMJ. 2020;370. 10.1136/bmj.m2815 32665317

[2] Barrett CE, Koyama AK, Alvarez P, Chow W, Lundeen EA, Perrine CG, . Risk for Newly Diagnosed Diabetes >30 Days After SARS-CoV-2 Infection Among Persons Aged <18 Years - United States, March 1, 2020–June 28, 2021. MMWR Morb Mortal Wkly Rep. 2022;71:59–65. doi: 10.15585/mmwr.mm7102e2. 35025851 PMC8757617

[3] Birabaharan M, Kaelber DC, Pettus JH, Smith DM. Risk of new-onset type 2 diabetes in 600 055 people after COVID-19: A cohort study. Diabetes Obes Metab. 2022;24:1176–9. doi: 10.1111/dom.14659. 35112782 PMC9035030

[4] Qeadan F, Tingey B, Egbert J, Pezzolesi MG, Burge MR, Peterson KA, . The associations between COVID-19 diagnosis, type 1 diabetes, and the risk of diabetic ketoacidosis: A nationwide cohort from the US using the Cerner Real-World Data. PLoS One. 2022;17:e0266809. doi: 10.1371/journal.pone.0266809. 35439266 PMC9017888

[5] Kendall EK, Olaker VR, Kaelber DC, Xu R, Davis PB. Association of SARS-CoV-2 Infection With New-Onset Type 1 Diabetes Among Pediatric Patients From 2020 to 2021. JAMA Netw Open. 2022;5:e2233014. doi: 10.1001/jamanetworkopen.2022.33014. 36149658 PMC9508649

[6] Ayoubkhani D, Khunti K, Nafilyan V, Maddox T, Humberstone B, Diamond I, . Post-covid syndrome in individuals admitted to hospital with covid-19: retrospective cohort study. BMJ. 2021;372:n693. doi: 10.1136/bmj.n693. 33789877 PMC8010267

[7] Wander PL, Lowy E, Beste LA, Tulloch-Palomino L, Korpak A, Peterson AC, . The Incidence of Diabetes Among 2,777,768 Veterans With and Without Recent SARS-CoV-2 Infection. Diabetes Care. 2022;45:782–8. doi: 10.2337/dc21-1686. 35085391 PMC9016731

[8] Xie Y, Al-Aly Z. Risks and burdens of incident diabetes in long COVID: a cohort study. Lancet Diabetes Endocrinol. 2022;10:311–21. doi: 10.1016/S2213-8587(22)00044-4. 35325624 PMC8937253

[9] Takashima S, Tokiya M, Izui K, Miyamoto H, Matsumoto A. Asian flush is a potential protective factor against COVID-19: a web-based retrospective survey in Japan. Environ Health Prev Med. 2024;29:14. doi: 10.1265/ehpm.23-00361. 38462476 PMC10937249

[10] Namkoong H, Edahiro R, Takano T, Nishihara H, Shirai Y, Sonehara K, . DOCK2 is involved in the host genetics and biology of severe COVID-19. Nature. 2022;609:754–60. doi: 10.1038/s41586-022-05163-5. 35940203 PMC9492544

[11] Accili D. Can COVID-19 cause diabetes? Nat Metab. 2021;3:123–5. doi: 10.1038/s42255-020-00339-7. 33432203 PMC8892570

[12] Tang X, Uhl S, Zhang T, Xue D, Li B, Vandana JJ, . SARS-CoV-2 infection induces beta cell transdifferentiation. Cell Metab. 2021;33:1577–91 e7. doi: 10.1016/j.cmet.2021.05.015. 34081913 PMC8133495

[13] Zheng C, Shao W, Chen X, Zhang B, Wang G, Zhang W. Real-world effectiveness of COVID-19 vaccines: a literature review and meta-analysis. Int J Infect Dis. 2022;114:252–60. doi: 10.1016/j.ijid.2021.11.009. 34800687 PMC8595975

[14] Japanese Ministry of Health Labour and Welfare https://www.mhlw.go.jp/stf/seisakunitsuite/bunya/newpage_00045.html (2023).

