# Supplementary material for: Differential impact of SARS-CoV-2 infection during different outbreak periods on incident diabetes in Japan: a matched cohort study utilizing health insurance claims
Source: Environ Health Prev Med. 2024 Oct 3;29:52. doi: 10.1265/ehpm.24-00191 (PMC11473386; doi:10.1265/ehpm.24-00191)
Supplement: Supplementary file 1 — Additional file 1: Figure S1. Summary of publicly available statistics in Japan on (a) sequences by viral variant of SARS-CoV-2 and (b) rate of COVID-19 vaccination. Figure S2. Plots of log (−log (day of diabetes-free survival)) versus log (diabetes-free survival ratio). Figure S3. Type 2 diabetes-free survival curves by COVID-19 exposure stratified by (a) sex and (b) age. Table S1. Incident type 1 diabetes in COVID-19 exposed and non-exposed cohorts during 2-month follow-up. Table S2. Incident type 2 diabetes in COVID-19 exposed and non-exposed cohorts during 1-year follow-up. Table S3. Hazard ratio estimation for type 2 diabetes by exposure to the first COVID-19 with 2-month follow-up. Table S4. Hazard ratio estimation for type 2 diabetes by exposure to the first COVID-19 with 1-year follow-up. [file ehpm-29-052-s001.docx]

Supplementary Material

Figure S1. Summary of publicly available statistics in Japan on (a) sequences by viral variant of SARS-CoV-2 (Tokyo Metropolitan Government Bureau of Public Health https://www.hokeniryo.metro.tokyo.lg.jp/index.html) and (b) rate of COVID-19 vaccination (digital Agency Vaccination Record System. Available online: https://info.vrs.digital.go.jp/opendata/).

Figure S2. Plots of log (-log (day of diabetes-free survival)) versus log (diabetes-free survival ratio). Hazard proportionality was tested by the parallelism between the curve of the COVID-19 exposed and unexposed cohorts to examine the suitability for the Cox proportional hazards model. The 60-day plot (left panel) shows better parallelism than the 3-year plot.

Figure S3. Type 2 diabetes-free survival curves by COVID-19 exposure stratified by (a) sex and (b) age.

Table S1. Incident type 1 diabetes in COVID-19 exposed and non-exposed cohorts during 2-month follow-up.

|  | I | | II | | III | | IV | | V | |
| --- | --- | --- | --- | --- | --- | --- | --- | --- | --- | --- |
|  | 2020 (April 2020 - March 2021) | | 4th (April - May 2021) | | 5th (July -September 2021) | | 6th (January - March 2022) | | 7th (July - September 2022) | |
|  | Exposed | Non-exposed | Exposed | Non-exposed | Exposed | Non-exposed | Exposed | Non-exposed | Exposed | Non-exposed |
|  | Cases/observations (%) | | Cases/observations (%) | | Cases/observations (%) | | Cases/observations (%) | | Cases/observations (%) | |
| All | 0/5,982 (0.00) | 0/23,928 (0.00) | 0/3,092 (0.00) | 1/12,368 (0.01) | 0/8,226 (0.00) | 0/32,904 (0.00) | 3/86,149 (0.00) | 4/344,596 (0.00) | 1/184,155 (0.00) | 9/736,620 (0.00) |
| <10 y.o. | 0/845 (0.00) | 0/3,380 (0.00) | 0/340 (0.00) | 0/1,360 (0.00) | 0/944 (0.00) | 0/3,776 (0.00) | 1/12,194 (0.01) | 0/48,776 (0.00) | 0/12,475 (0.00) | 1/49,900 (0.00) |
| 10–19 | 0/645 (0.00) | 0/2,580 (0.00) | 0/374 (0.00) | 0/1,496 (0.00) | 0/1,418 (0.00) | 0/5,672 (0.00) | 0/17,312 (0.00) | 0/69,248 (0.00) | 0/33,029 (0.00) | 1/132,116 (0.00) |
| 20–29 | 0/857 (0.00) | 0/3,428 (0.00) | 0/433 (0.00) | 0/1,732 (0.00) | 0/1,299 (0.00) | 0/5,196 (0.00) | 0/9,199 (0.00) | 0/36,796 (0.00) | 0/20,250 (0.00) | 0/81,000 (0.00) |
| 30–39 | 0/1,097 (0.00) | 0/4,388 (0.00) | 0/556 (0.00) | 0/2,224 (0.00) | 0/1,523 (0.00) | 0/6,092 (0.00) | 1/17,975 (0.01) | 0/71,900 (0.00) | 0/32,571 (0.00) | 0/130,284 (0.00) |
| 40–49 | 0/1,055 (0.00) | 0/4,220 (0.00) | 0/538 (0.00) | 0/2,152 (0.00) | 0/1,550 (0.00) | 0/6,200 (0.00) | 1/16,741 (0.01) | 1/66,964 (0.00) | 1/42,023 (0.00) | 4/168,092 (0.00) |
| 50–59 | 0/1,143 (0.00) | 0/4,572 (0.00) | 0/637 (0.00) | 0/2,548 (0.00) | 0/1,224 (0.00) | 0/4,896 (0.00) | 0/9,713 (0.00) | 3/38,852 (0.01) | 0/33,541 (0.00) | 2/134,164 (0.00) |
| ≥60 | 0/340 (0.00) | 0/1,360 (0.00) | 0/214 (0.00) | 1/856 (0.12) | 0/268 (0.00) | 0/1,072 (0.00) | 0/3,015 (0.00) | 0/12,060 (0.00) | 0/10,266 (0.00) | 1/41,064 (0.00) |
| Males | 0/3,202 (0.00) | 0/12,808 (0.00) | 0/1,597 (0.00) | 1/6,388 (0.02) | 0/4,355 (0.00) | 0/17,420 (0.00) | 0/40,355 (0.00) | 3/161,420 (0.00) | 1/85,355 (0.00) | 4/341,420 (0.00) |
| <10 y.o. | 0 /464 (0.00) | 0/1,856 (0.00) | 0/183 (0.00) | 0/732 (0.00) | 0/545 (0.00) | 0/2,180 (0.00) | 0/6,123 (0.00) | 0/24,492 (0.00) | 0/6,139 (0.00) | 0/24,556 (0.00) |
| 10–19 | 0 /345 (0.00) | 0/1,380 (0.00) | 0/213 (0.00) | 0/852 (0.00) | 0/763 (0.00) | 0/3,052 (0.00) | 0/9,542 (0.00) | 0/38,168 (0.00) | 0/15,912 (0.00) | 1/63,648 (0.00) |
| 20–29 | 0 /410 (0.00) | 0/1,640 (0.00) | 0/200 (0.00) | 0/800 (0.00) | 0/653 (0.00) | 0/2,612 (0.00) | 0/4,263 (0.00) | 0/17,052 (0.00) | 0/9,043 (0.00) | 0/36,172 (0.00) |
| 30–39 | 0 /550 (0.00) | 0/2,200 (0.00) | 0/256 (0.00) | 0/1,024 (0.00) | 0/752 (0.00) | 0/3,008 (0.00) | 0/7,437 (0.00) | 0/29,748 (0.00) | 0/13,895 (0.00) | 0/55,580 (0.00) |
| 40–49 | 0/551 (0.00) | 0/2,204 (0.00) | 0/267 (0.00) | 0/1,068 (0.00) | 0/792 (0.00) | 0/3,168 (0.00) | 0/6,762 (0.00) | 1/27,048 (0.00) | 1/18,507 (0.01) | 2/74,028 (0.00) |
| 50–59 | 0/661 (0.00) | 0/2,644 (0.00) | 0/351 (0.00) | 0/1,404 (0.00) | 0/677 (0.00) | 0/2,708 (0.00) | 0/4,579 (0.00) | 2/18,316 (0.01) | 0/16,288 (0.00) | 1/65,152 (0.00) |
| ≥60 | 0/221 (0.00) | 0/884 (0.00) | 0/127 (0.00) | 1/508 (0.20) | 0/173 (0.00) | 0/692 (0.00) | 0/1,649 (0.00) | 0/6,596 (0.00) | 0/5,571 (0.00) | 0/22,284 (0.00) |
| Females | 0/2,780 (0.00) | 0/11,120 (0.00) | 0/1,495 (0.00) | 0/5,980 (0.00) | 0/3,871 (0.00) | 0/15,484 (0.00) | 3/45,794 (0.01) | 1/183,176 (0.00) | 0/98,800 (0.00) | 5/395,200 (0.00) |
| <10 y.o. | 0/381 (0.00) | 0/1,524 (0.00) | 0/157 (0.00) | 0/628 (0.00) | 0/399 (0.00) | 0/1,596 (0.00) | 1/6,071 (0.02) | 0/24,284 (0.00) | 0/6,336 (0.00) | 1/25,344 (0.00) |
| 10–19 | 0/300 (0.00) | 0/1,200 (0.00) | 0/161 (0.00) | 0/644 (0.00) | 0/655 (0.00) | 0/2,620 (0.00) | 0/7,770 (0.00) | 0/31,080 (0.00) | 0/17,117 (0.00) | 0/68,468 (0.00) |
| 20–29 | 0/447 (0.00) | 0/1,788 (0.00) | 0/233 (0.00) | 0/932 (0.00) | 0/646 (0.00) | 0/2,584 (0.00) | 0/4,936 (0.00) | 0/19,744 (0.00) | 0/11,207 (0.00) | 0/44,828 (0.00) |
| 30–39 | 0/547 (0.00) | 0/2,188 (0.00) | 0/300 (0.00) | 0/1,200 (0.00) | 0/771 (0.00) | 0/3,084 (0.00) | 1/10,538 (0.01) | 0/42,152 (0.00) | 0/18,676 (0.00) | 0/74,704 (0.00) |
| 40–49 | 0/504 (0.00) | 0/2,016 (0.00) | 0/271 (0.00) | 0/1,084 (0.00) | 0/758 (0.00) | 0/3,032 (0.00) | 1/9,979 (0.01) | 0/39,916 (0.00) | 0/23,516 (0.00) | 2/94,064 (0.00) |
| 50–59 | 0/482 (0.00) | 0/1,928 (0.00) | 0/286 (0.00) | 0/1,144 (0.00) | 0/547 (0.00) | 0/2,188 (0.00) | 0/5,134 (0.00) | 1/20,536 (0.00) | 0/17,253 (0.00) | 1/69,012 (0.00) |
| ≥60 | 0/119 (0.00) | 0/476 (0.00) | 0/87 (0.00) | 0/348 (0.00) | 0/95 (0.00) | 0/380 (0.00) | 0/1,366 (0.00) | 0/5,464 (0.00) | 0/4,695 (0.00) | 1/18,780 (0.01) |

Table S2. Incident type 2 diabetes in COVID-19 exposed and non-exposed cohorts during 1-year follow-up.

|  | I | | II | | III | | IV | | V* | |
| --- | --- | --- | --- | --- | --- | --- | --- | --- | --- | --- |
|  | 2020 (April 2020 - March 2021) | | 4th (April - May 2021) | | 5th (July -September 2021) | | 6th (January - March 2022) | | 7th (July - September 2022) | |
|  | Exposed | Non-exposed | Exposed | Non-exposed | Exposed | Non-exposed | Exposed | Non-exposed | Exposed | Non-exposed |
|  | Cases/observations (%) | | Cases/observations (%) | | Cases/observations (%) | | Cases/observations (%) | | Cases/observations (%) | |
| All | 121/5,982 (2.02) | 138/23,928 (0.58) | 77/3,092 (2.49) | 69/12,368 (0.56) | 157/8,226 (1.91) | 177/32,904 (0.54) | 465/86,149 (0.54) | 1468/344,596 (0.43) | 424/184,155 (0.23) | 1655/736,620 (0.22) |
| <10 y.o. | 0/845 (0.00) | 0/3,380 (0.00) | 0/340 (0.00) | 0/1,360 (0.00) | 0/944 (0.00) | 2/3,776 (0.05) | 1/12,194 (0.01) | 3/48,776 (0.01) | 2/12,475 (0.02) | 1/49,900 (0.00) |
| 10–19 | 3/645 (0.47) | 1/2,580 (0.04) | 1/374 (0.27) | 1/1,496 (0.07) | 1/1,418 (0.07) | 0/5,672 (0.00) | 14/17,312 (0.08) | 38/69,248 (0.05) | 3/33,029 (0.01) | 26/132,116 (0.02) |
| 20–29 | 2/857 (0.23) | 11/3,428 (0.32) | 1/433 (0.23) | 3/1,732 (0.17) | 7/1,299 (0.54) | 11/5,196 (0.21) | 25/9,199 (0.27) | 67/36,796 (0.18) | 10/20,250 (0.05) | 53/81,000 (0.07) |
| 30–39 | 9/1,097 (0.82) | 21/4,388 (0.48) | 9/556 (1.62) | 9/2,224 (0.40) | 26/1,523 (1.71) | 25/6,092 (0.41) | 71/17,975 (0.39) | 262/71,900 (0.36) | 44/32,571 (0.14) | 203/130,284 (0.16) |
| 40–49 | 31/1,055 (2.94) | 30/4,220 (0.71) | 14/538 (2.60) | 13/2,152 (0.60) | 47/1,550 (3.03) | 54/6,200 (0.87) | 136/16,741 (0.81) | 466/66,964 (0.70) | 112/42,023 (0.27) | 496/168,092 (0.30) |
| 50–59 | 57/1,143 (4.99) | 55/4,572 (1.20) | 40/637 (6.28) | 26/2,548 (1.02) | 58/1,224 (4.74) | 65/4,896 (1.33) | 143/9,713 (1.47) | 475/38,852 (1.22) | 172/33,541 (0.51) | 647/134,164 (0.48) |
| ≥60 | 19/340 (5.59) | 20/1,360 (1.47) | 12/214 (5.61) | 17/856 (1.99) | 18/268 (6.72) | 20/1,072 (1.87) | 75/3,015 (2.49) | 157/12,060 (1.30) | 81/10,266 (0.79) | 229/41,064 (0.56) |
|  |  |  |  |  |  |  |  |  |  |  |
| Males | 91/3,202 (2.84) | 90/12,808 (0.70) | 54/1,597 (3.38) | 44/6,388 (0.69) | 113/4,355 (2.59) | 116/17,420 (0.67) | 261/40,355 (0.65) | 830/161,420 (0.51) | 265/85,355 (0.31) | 1015/341,420 (0.30) |
| <10 y.o. | 0/464 (0.00) | 0/1,856 (0.00) | 0/183 (0.00) | 0/732 (0.00) | 0/545 (0.00) | 0/2,180 (0.00) | 0/6,123 (0.00) | 0/24,492 (0.00) | 2/6,139 (0.03) | 0/24,556 (0.00) |
| 10–19 | 1/345 (0.29) | 0/1,380 (0.00) | 1/213 (0.47) | 1/852 (0.12) | 1/763 (0.13) | 0/3,052 (0.00) | 8/9,542 (0.08) | 20/38,168 (0.05) | 1/15,912 (0.01) | 14/63,648 (0.02) |
| 20–29 | 0/410 (0.00) | 5/1,640 (0.30) | 0/200 (0.00) | 3/800 (0.38) | 3/653 (0.46) | 8/2,612 (0.31) | 7/4,263 (0.16) | 38/17,052 (0.22) | 4/9,043 (0.04) | 31/36,172 (0.09) |
| 30–39 | 8/550 (1.45) | 12/2,200 (0.55) | 6/256 (2.34) | 3/1,024 (0.29) | 17/752 (2.26) | 13/3,008 (0.43) | 36/7,437 (0.48) | 131/29,748 (0.44) | 18/13,895 (0.13) | 116/55,580 (0.21) |
| 40–49 | 23/551 (4.17) | 23/2,204 (1.04) | 8/267 (3.00) | 7/1,068 (0.66) | 40/792 (5.05) | 34/3,168 (1.07) | 70/6,762 (1.04) | 258/27,048 (0.95) | 72/18,507 (0.39) | 295/74,028 (0.40) |
| 50–59 | 43/661 (6.51) | 39/2,644 (1.48) | 32/351 (9.12) | 19/1,404 (1.35) | 38/677 (5.61) | 46/2,708 (1.70) | 84/4,579 (1.83) | 287/18,316 (1.57) | 112/16,288 (0.69) | 415/65,152 (0.64) |
| ≥60 | 16/221 (7.24) | 11/884 (1.24) | 7/127 (5.51) | 11/508 (2.17) | 14/173 (8.09) | 15/692 (2.17) | 56/1,649 (3.40) | 96/6,596 (1.46) | 56/5,571 (1.01) | 144/22,284 (0.65) |
|  |  |  |  |  |  |  |  |  |  |  |
| Females | 30/2,780 (1.08) | 48/11,120 (0.43) | 23/1,495 (1.54) | 25/5,980 (0.42) | 44/3,871 (1.14) | 61/15,484 (0.39) | 204/45,794 (0.45) | 638/183,176 (0.35) | 159/98,800 (0.16) | 640/395,200 (0.16) |
| <10 y.o. | 0/381 (0.00) | 0/1,524 (0.00) | 0/157 (0.00) | 0/628 (0.00) | 0/399 (0.00) | 2/1,596 (0.13) | 1/6,071 (0.02) | 3/24,284 (0.01) | 0/6,336 (0.00) | 1/25,344 (0.00) |
| 10–19 | 2/300 (0.67) | 1/1,200 (0.08) | 0/161 (0.00) | 0/644 (0.00) | 0/655 (0.00) | 0/2,620 (0.00) | 6/7,770 (0.08) | 18/31,080 (0.06) | 2/17,117 (0.01) | 12/68,468 (0.02) |
| 20–29 | 2/447 (0.45) | 6/1,788 (0.34) | 1/233 (0.43) | 0/932 (0.00) | 4/646 (0.62) | 3/2,584 (0.12) | 18/4,936 (0.36) | 29/19,744 (0.15) | 6/11,207 (0.05) | 22/44,828 (0.05) |
| 30–39 | 1/547 (0.18) | 9/2,188 (0.41) | 3/300 (1.00) | 6/1,200 (0.50) | 9/771 (1.17) | 12/3,084 (0.39) | 35/10,538 (0.33) | 131/42,152 (0.31) | 26/18,676 (0.14) | 87/74,704 (0.12) |
| 40–49 | 8/504 (1.59) | 7/2,016 (0.35) | 6/271 (2.21) | 6/1,084 (0.55) | 7/758 (0.92) | 20/3,032 (0.66) | 66/9,979 (0.66) | 208/39,916 (0.52) | 40/23,516 (0.17) | 201/94,064 (0.21) |
| 50–59 | 14/482 (2.90) | 16/1,928 (0.83) | 8/286 (2.80) | 7/1,144 (0.61) | 20/547 (3.66) | 19/2,188 (0.87) | 59/5,134 (1.15) | 188/20,536 (0.92) | 60/17,253 (0.35) | 232/69,012 (0.34) |
| ≥60 | 3/119 (2.52) | 9/476 (1.89) | 5/87 (5.75) | 6/348 (1.72) | 4/95 (4.21) | 5/380 (1.32) | 19/1,366 (1.39) | 61/5,464 (1.12) | 25/4,695 (0.53) | 85/18,780 (0.45) |

*Observation ended on March 31, 2023.

Table S3. Hazard ratio estimation for type 2 diabetes by exposure to the first COVID-19 with 2-month follow-up.

|  |  | I | | | | | II | | | | | III | | | | | IV | | | | | V | | | | |
| --- | --- | --- | --- | --- | --- | --- | --- | --- | --- | --- | --- | --- | --- | --- | --- | --- | --- | --- | --- | --- | --- | --- | --- | --- | --- | --- |
|  |  | 2020 (April 2020 - March 2021) | | | | | 4th (April - May 2021) | | | | | 5th (July -September 2021) | | | | | 6th (January - March 2022) | | | | | 7th (July - September 2022) | | | | |
|  |  | HR | 95%CI | | | p-value | HR | 95%CI | | | p-value | HR | 95%CI | | | p-value | HR | 95%CI | | | p-value | HR | 95%CI | | | p-value |
| All | Crude Model | 13.99 | 8.61 | - | 22.73 | < 0.001 | 15.86 | 8.82 | - | 28.52 | < 0.001 | 19.83 | 12.90 | - | 30.49 | < 0.001 | 1.99 | 1.60 | - | 2.48 | < 0.001 | 1.01 | 0.86 | - | 1.19 | 0.908 |
|  | Model 1 | 13.77 | 8.53 | - | 22.26 | < 0.001 | 16.61 | 9.23 | - | 29.87 | < 0.001 | 20.88 | 13.58 | - | 32.10 | < 0.001 | 2.06 | 1.65 | - | 2.56 | < 0.001 | 1.03 | 0.88 | - | 1.21 | 0.693 |
|  | Model 2 | 14.17 | 8.71 | - | 23.05 | < 0.001 | 16.71 | 9.29 | - | 30.06 | < 0.001 | 20.80 | 13.52 | - | 31.99 | < 0.001 | 2.02 | 1.62 | - | 2.52 | < 0.001 | 1.02 | 0.87 | - | 1.19 | 0.847 |
| Males | Crude Model | 18.47 | 10.36 | - | 32.93 | < 0.001 | 15.79 | 7.88 | - | 31.64 | < 0.001 | 22.36 | 13.34 | - | 37.48 | < 0.001 | 2.05 | 1.53 | - | 2.75 | < 0.001 | 1.02 | 0.83 | - | 1.25 | 0.849 |
|  | Model 1 | 19.08 | 10.70 | - | 34.02 | < 0.001 | 44.68 | 17.11 | - | 116.69 | < 0.001 | 23.60 | 14.08 | - | 39.56 | < 0.001 | 2.12 | 1.58 | - | 2.85 | < 0.001 | 1.05 | 0.85 | - | 1.28 | 0.677 |
|  | Model 2 | 18.72 | 10.49 | - | 33.43 | < 0.001 | 45.17 | 17.21 | - | 118.54 | < 0.001 | 23.46 | 13.99 | - | 39.35 | < 0.001 | 2.07 | 1.54 | - | 2.78 | < 0.001 | 1.03 | 0.84 | - | 1.26 | 0.787 |
| Females | Crude Model | 5.15 | 1.92 | - | 13.84 | 0.001 | 16.09 | 5.38 | - | 48.13 | < 0.001 | 14.56 | 6.66 | - | 31.84 | < 0.001 | 1.93 | 1.39 | - | 2.68 | < 0.001 | 0.99 | 0.77 | - | 1.28 | 0.958 |
|  | Model 1 | 5.18 | 1.93 | - | 13.92 | 0.001 | 17.32 | 5.78 | - | 51.87 | < 0.001 | 15.15 | 6.92 | - | 33.15 | < 0.001 | 1.98 | 1.43 | - | 2.75 | < 0.001 | 1.02 | 0.79 | - | 1.31 | 0.908 |
|  | Model 2 | 5.21 | 1.94 | - | 14.01 | 0.001 | 17.32 | 5.78 | - | 51.87 | < 0.001 | 15.20 | 6.95 | - | 33.26 | < 0.001 | 1.97 | 1.42 | - | 2.73 | < 0.001 | 1.00 | 0.77 | - | 1.29 | 0.973 |
| <10 y.o | Crude Model | - | - |  | - | - | - | - |  | - | - | - | - |  | - | - | 4.00 | 0.56 | - | 28.40 | 0.166 | 4.00 | 0.56 | - | 28.40 | 0.166 |
|  | Model 1 | - | - |  | - | - | - | - |  | - | - | - | - |  | - | - | 2.85 | 0.39 | - | 21.05 | 0.306 | 3.84 | 0.54 | - | 27.50 | 0.181 |
|  | Model 2 | - | - |  | - | - | - | - |  | - | - | - | - |  | - | - | 2.85 | 0.39 | - | 21.06 | 0.305 | 3.84 | 0.54 | - | 27.55 | 0.181 |
| 10–19 | Crude Model | - | - |  | - | - | - | - |  | - | - | - | - |  | - | - | 4.00 | 0.81 | - | 19.82 | 0.09 | 0.47 | 0.11 | - | 2.04 | 0.313 |
|  | Model 1 | - | - |  | - | - | - | - |  | - | - | - | - |  | - | - | 1.67 | 0.28 | - | 10.14 | 0.575 | 0.51 | 0.12 | - | 2.20 | 0.364 |
|  | Model 2 | - | - |  | - | - | - | - |  | - | - | - | - |  | - | - | 1.68 | 0.28 | - | 10.14 | 0.575 | 0.51 | 0.12 | - | 2.20 | 0.364 |
| 20–29 | Crude Model | 4.00 | 0.25 | - | 63.99 | 0.327 | - | - |  | - | - | 16.01 | 1.79 | - | 143.27 | 0.013 | 3.50 | 1.27 | - | 9.66 | 0.015 | 0.46 | 0.14 | - | 1.53 | 0.205 |
|  | Model 1 | 4.58 | 0.29 | - | 73.65 | 0.283 | - | - |  | - | - | 19.46 | 2.09 | - | 181.39 | 0.009 | 3.47 | 1.26 | - | 9.58 | 0.016 | 0.45 | 0.14 | - | 1.46 | 0.184 |
|  | Model 2 | 4.58 | 0.28 | - | 73.65 | 0.283 | - | - |  | - | - | 19.46 | 2.09 | - | 181.39 | 0.009 | 3.48 | 1.26 | - | 9.59 | 0.016 | 0.45 | 0.14 | - | 1.46 | 0.186 |
| 30–39 | Crude Model | 12.03 | 2.43 | - | 59.61 | 0.002 | 3.01 | 0.67 | - | 13.45 | 0.149 | 11.39 | 4.49 | - | 28.89 | < 0.001 | 1.54 | 0.85 | - | 2.79 | 0.156 | 0.79 | 0.46 | - | 1.35 | 0.389 |
|  | Model 1 | 14.18 | 2.79 | - | 72.11 | 0.001 | 4.20 | 0.89 | - | 19.90 | 0.071 | 12.01 | 4.72 | - | 30.58 | < 0.001 | 1.53 | 0.85 | - | 2.78 | 0.160 | 0.79 | 0.46 | - | 1.36 | 0.395 |
|  | Model 2 | 14.24 | 2.80 | - | 72.38 | 0.001 | 4.20 | 0.89 | - | 19.89 | 0.071 | 12.00 | 4.72 | - | 30.54 | < 0.001 | 1.53 | 0.85 | - | 2.78 | 0.159 | 0.78 | 0.46 | - | 1.34 | 0.365 |
| 40–49 | Crude Model | 9.58 | 4.20 | - | 21.89 | < 0.001 | 22.2 | 4.93 | - | 100.25 | < 0.001 | 43.57 | 15.64 | - | 121.39 | < 0.001 | 1.32 | 0.85 | - | 2.05 | 0.223 | 0.92 | 0.67 | - | 1.26 | 0.599 |
|  | Model 1 | 10.28 | 4.49 | - | 23.57 | < 0.001 | 7.88 | 2.49 | - | 25.01 | < 0.001 | 47.06 | 16.90 | - | 131.06 | < 0.001 | 1.35 | 0.86 | - | 2.10 | 0.193 | 0.92 | 0.67 | - | 1.26 | 0.591 |
|  | Model 2 | 10.36 | 4.52 | - | 23.76 | < 0.001 | 7.89 | 2.49 | - | 25.05 | < 0.001 | 47.07 | 16.90 | - | 131.07 | < 0.001 | 1.33 | 0.85 | - | 2.09 | 0.207 | 0.90 | 0.66 | - | 1.24 | 0.529 |
| 50–59 | Crude Model | 18.30 | 8.51 | - | 39.38 | < 0.001 | 20.49 | 8.53 | - | 49.22 | < 0.001 | 17.45 | 9.05 | - | 33.65 | < 0.001 | 2.25 | 1.57 | - | 3.23 | < 0.001 | 1.09 | 0.85 | - | 1.40 | 0.484 |
|  | Model 1 | 19.27 | 8.94 | - | 41.51 | < 0.001 | 22.14 | 9.20 | - | 53.31 | < 0.001 | 17.63 | 9.14 | - | 34.01 | < 0.001 | 2.28 | 1.59 | - | 3.27 | < 0.001 | 1.11 | 0.87 | - | 1.42 | 0.407 |
|  | Model 2 | 19.09 | 8.85 | - | 41.20 | < 0.001 | 22.30 | 9.26 | - | 53.70 | < 0.001 | 17.67 | 9.16 | - | 34.09 | < 0.001 | 2.22 | 1.55 | - | 3.20 | < 0.001 | 1.10 | 0.86 | - | 1.41 | 0.467 |
| ≥60 | Crude Model | 22.33 | 4.95 | - | 100.73 | < 0.001 | 18.35 | 3.96 | - | 84.92 | < 0.001 | 16.38 | 4.62 | - | 58.05 | < 0.001 | 3.01 | 1.71 | - | 5.30 | < 0.001 | 1.40 | 0.96 | - | 2.04 | 0.083 |
|  | Model 1 | 23.62 | 5.20 | - | 107.37 | < 0.001 | 32.14 | 6.12 | - | 168.85 | < 0.001 | 20.55 | 5.49 | - | 76.93 | < 0.001 | 3.11 | 1.76 | - | 5.49 | < 0.001 | 1.40 | 0.95 | - | 2.04 | 0.085 |
|  | Model 2 | 21.71 | 4.72 | - | 99.73 | < 0.001 | 32.10 | 6.12 | - | 168.45 | < 0.001 | 58.13 | 7.71 | - | 438.34 | < 0.001 | 2.95 | 1.66 | - | 5.24 | < 0.001 | 1.35 | 0.92 | - | 1.98 | 0.126 |

Model 1 includes age group, sex, residential area, and price range of healthcare receipt. Model 2 additionally contains a time-dependent covariate, days to hospitalization.

Table S4. Hazard ratio estimation for type 2 diabetes by exposure to the first COVID-19 with 1-year follow-up.

|  | I | | | | | | II | | | | | III | | | | | IV | | | | | V* | | | | |
| --- | --- | --- | --- | --- | --- | --- | --- | --- | --- | --- | --- | --- | --- | --- | --- | --- | --- | --- | --- | --- | --- | --- | --- | --- | --- | --- |
|  | 2020 (April 2020 – March 2021) | | | | | | 4^th^ (April – May 2021) | | | | | 5^th^ (July -September 2021) | | | | | 6^th^ (January – March 2022) | | | | | 7^th^ (July – September 2022) | | | | |
|  | HR | 95%CI | | | p-value | HR | | 95%CI | | | p-value | HR | 95%CI | | | p-value | HR | 95%CI | | | p-value | HR | 95%CI | | | p-value |
| All |  |  |  |  |  |  | |  |  |  |  |  |  |  |  |  |  |  |  |  |  |  |  |  |  |  |
| Crude Model | 3.52 | 2.76 | - | 4.49 | < 0.001 | 4.52 | | 3.27 | - | 6.24 | < 0.001 | 3.51 | 2.84 | - | 4.35 | < 0.001 | 1.27 | 1.14 | - | 1.40 | < 0.001 | 1.04 | 0.95 | - | 1.14 | 0.422 |
| Model 1 | 3.61 | 2.83 | - | 4.60 | < 0.001 | 4.74 | | 3.43 | - | 6.55 | < 0.001 | 3.68 | 2.98 | - | 4.56 | < 0.001 | 1.30 | 1.18 | - | 1.45 | < 0.001 | 1.06 | 0.96 | - | 1.17 | 0.230 |
| Model 2 | 3.53 | 2.76 | - | 4.51 | < 0.001 | 4.72 | | 3.41 | - | 6.52 | < 0.001 | 3.67 | 2.96 | - | 4.54 | < 0.001 | 1.30 | 1.17 | - | 1.44 | < 0.001 | 1.05 | 0.96 | - | 1.16 | 0.281 |
| Males |  |  |  |  |  |  | |  |  |  |  |  |  |  |  |  |  |  |  |  |  |  |  |  |  |  |
| Crude Model | 4.16 | 3.11 | - | 5.56 | < 0.001 | 5.00 | | 3.36 | - | 7.45 | < 0.001 | 3.83 | 2.96 | - | 4.95 | < 0.001 | 1.25 | 1.09 | - | 1.44 | 0.002 | 1.05 | 0.93 | - | 1.19 | 0.418 |
| Model 1 | 4.30 | 3.21 | - | 5.75 | < 0.001 | 5.25 | | 3.52 | - | 7.82 | < 0.001 | 4.05 | 3.13 | - | 5.24 | < 0.001 | 1.29 | 1.13 | - | 1.49 | < 0.001 | 1.07 | 0.94 | - | 1.20 | 0.313 |
| Model 2 | 4.18 | 3.12 | - | 5.60 | < 0.001 | 5.25 | | 3.52 | - | 7.83 | < 0.001 | 4.02 | 3.11 | - | 5.20 | < 0.001 | 1.29 | 1.12 | - | 1.48 | < 0.001 | 1.06 | 0.94 | - | 1.20 | 0.320 |
| Females |  |  |  |  |  |  | |  |  |  |  |  |  |  |  |  |  |  |  |  |  |  |  |  |  |  |
| Crude Model | 2.41 | 1.53 | - | 3.79 | < 0.001 | 3.73 | | 2.14 | - | 6.49 | < 0.001 | 2.92 | 1.99 | - | 4.29 | < 0.001 | 1.29 | 1.10 | - | 1.50 | 0.002 | 1.02 | 0.87 | - | 1.19 | 0.809 |
| Model 1 | 2.42 | 1.54 | - | 3.80 | < 0.001 | 3.98 | | 2.28 | - | 6.93 | < 0.001 | 3.01 | 2.05 | - | 4.41 | < 0.001 | 1.32 | 1.13 | - | 1.54 | < 0.001 | 1.06 | 0.90 | - | 1.23 | 0.498 |
| Model 2 | 2.40 | 1.52 | - | 3.78 | < 0.001 | 3.92 | | 2.24 | - | 6.88 | < 0.001 | 3.03 | 2.06 | - | 4.44 | < 0.001 | 1.31 | 1.12 | - | 1.54 | < 0.001 | 1.04 | 0.89 | - | 1.21 | 0.638 |

Model 1 includes age group, sex, residential area, and price range of healthcare receipt. Model 2 additionally contains a time-dependent covariate, days to hospitalization. *Observation ended on March 31, 2023.
